# Probabilistic classifier: generated using randomised sub-sampling of the feature space

**DOI:** 10.1186/1758-2946-4-S1-P40

**Published:** 2012-05-01

**Authors:** Jonathan D Tyzack, Hamse Y Mussa, Robert C Glen

**Affiliations:** 1 Unilever Centre for Molecular Sciences Informatics, Department of Chemistry, University of Cambridge, Cambridge, CB2 1EW, UK

## 

Nowadays supervised classification, based on the concept of pattern recognition, is an integral part of virtual screening. The central idea of supervised classification in chemoinformatics is to design a classifying algorithm that accurately assigns a new molecule to one of a set of predefined classes.

Naturally, probabilistic classifiers can be far more useful than hard point classifiers in making a decision on problems [1], such as virtual screening, where there is an associated risk in classifying an instance to one class or the other.

For their conceptual simplicity and computational efficiency probabilistic classification methods based on the Naive Bayes concept are widely employed in chemoinformatics. The simplicity of the Naive Bayes is due to the assumption that the descriptors representing the molecule one desires to classify are statistically independent. Unfortunately it is well documented that when the molecular descriptors are binary-valued - which is often the case in chemoinformatics - and thus take values of 0 or 1 the Naive Bayesian classifier can only act as a linear classifier in the descriptor space.

Techniques such as the Parzen-Window approach can address the above shortcomings but suffer from being computationally expensive as they require one to retain all the training dataset in core memory [2,3].

In an attempt to address the above mentioned drawbacks, a new probabilistic classifier is proposed which uses randomized sub-sampling of the descriptor space. The proposed algorithm generates better class membership predictions than its Naive Bayesian counterpart on classifying molecules that are non-linearly separable in descriptor space.

We present a realistic test of the new method by classifying large chemical datasets generated from the ChEMBL database [4].
